# A Review of Environmental Control Strategies and Models for Modern Agricultural Greenhouses

**DOI:** 10.3390/s25051388

**Published:** 2025-02-25

**Authors:** Shuailiang Chen, Aolong Liu, Fei Tang, Pei Hou, Yanli Lu, Pei Yuan

**Affiliations:** College New Energy, Zhengzhou University of Light Industry, Zhengzhou 450002, China

**Keywords:** control strategies, greenhouse models, modern agricultural greenhouse

## Abstract

As crucial sites for optimizing crop growth conditions, greenhouses have gained increasing favor among scholars due to their potential to significantly enhance food production. Greenhouse control involves regulating environmental parameters such as temperature, humidity, light, and CO_2_ concentration to ensure an optimal growth environment for crops while conserving energy. This paper provides an overview of various strategies for controlling greenhouse environments, encompassing structural control, environmental parameter management, and control algorithms, and points out that the integration of artificial neural networks with various optimization algorithms is a future trend. Additionally, it delves into the exploration of greenhouse microclimate models and crop growth models, noting that current models focus on some of the internal environmental parameters and that the models rely on empirical parameters. Therefore, multi-scale coupling of greenhouse models is the way forward. Furthermore, it provides insights into how to achieve sustainable energy use in greenhouses, and the application of digital twin technology in greenhouses is promising.

## 1. Introduction

Food is fundamental to human existence, and improving crop yield plays a role in human support. Greenhouses, as novel cultivation spaces, provide favorable conditions for plant growth and contribute to food production. According to statistics, there are approximately 3.64 million hectares of greenhouses globally [1]. As the area of greenhouses increases, the problem of high energy consumption of greenhouses is becoming increasingly serious. Greenhouse energy consumption includes heating, cooling, ventilation, and related aspects. Research has shown that controlling these factors and developing control strategies can improve greenhouse energy efficiency and reduce greenhouse energy consumption [2]. Therefore, research on greenhouse control strategies is essential for greenhouse agriculture.

The greenhouse system is a complex system with multi-input, multi-output, and mutual coupling. Environmental factors that affect crop growth and development in a greenhouse environment include temperature, humidity, light intensity, and carbon dioxide concentration. The purpose of greenhouse control is to enhance crop growth, improve yields, and maximize energy efficiency by regulating environmental parameters. Developing an efficient greenhouse control system requires starting with a greenhouse control strategy and synthesizing multiple factors. Strategies for controlling greenhouse conditions include structural control, environmental parameter control, control algorithm exploration, and greenhouse model research. Greenhouse structural control includes shading systems [3], ventilation systems [4,5], and refrigeration/heating systems [6]. Environmental parameter control in greenhouses mainly involves temperature control [7,8], humidity control [9,10], light modulation [11], CO_2_ concentration adjustment [12,13], airflow management [14,15], etc. Due to their significant and manageable impact on crop growth, most emphasis in environmental parameter control lies in regulating temperature and humidity within the greenhouse [16].

The developmental progression of control algorithms encompasses the following: early Proportional–Integral–Derivative (PID) control [17], regulating single factors only; fuzzy control [18] algorithms, enabling the control of multiple factors and variables within greenhouse environments; and feedback control [19], which achieves effective control performance through modeling. Adaptive control [20] shows outstanding control performance in the face of variations within greenhouse environments. Model predictive control [21] accurately predicts system behavior by setting precise mathematical models, enabling refined control. Neural network control [22,23], when applied to greenhouse regulation, reduces the need for making mathematical models, learning, predicting future behaviors from data, and achieving the best control effects. Hybrid control [24] combines diverse advantages to meet varied control requisites.

With the increasing concerns surrounding food security [25], greenhouse modeling has increasingly become a focal point. Greenhouse models mainly comprise the greenhouse microclimate model and crop growth model. The greenhouse microclimate models are mainly categorized as mechanical models and black-box models [26]. Based on the diverse methods of model establishment, their modeling tools are divided into artificial neural networks (ANNs) [27], computational fluid dynamics (CFD) [28], and other simulation software [29]. Crop growth models [30] quantitatively describe the dynamic processes of crop growth, development, seed formation, and yield under meteorological conditions, soil conditions, and crop management practices. Common crop growth models include the Decision Support System for Agrotechnology Transfer (DSSAT) model [31], Gossypium Simulation (GOSSYM) model [32], Crop Environment Resource Synthesis (CERES) model [33], Crop Growth (CROPGRO) model [34], Agricultural Production Systems Simulator (APSIM) model [35], and World Food Studies (WOFOST) model [36]. Both greenhouse microclimate and crop growth models have limited predictive accuracy in complex environments. The challenge of coupling these models and improving their precision in practical applications remains unresolved.

This study investigates the recent advancements in greenhouse control strategies and models. Greenhouse control can be classified into three main domains: structural control, parameter control, and control algorithms. The control algorithms are further categorized and detailed in this research. The third chapter provides a comprehensive overview of current greenhouse structural and parameter controls alongside a classification and explanation of control algorithms. The fourth chapter focuses on greenhouse microclimate models and crop growth models, classifying the former and summarizing the latter based on simulation tools. The last chapter summarizes existing strategies and models for greenhouse environmental control and outlines future directions for greenhouse control and modeling. It is anticipated that this review will serve as a reference for research on greenhouse environmental studies in China.

## 2. Acquisition of Document

This study employed a systematic literature review method, conducting searches across Web of Science, Science Direct, Engineering Village, Scopus, and Google Scholar databases for relevant publications spanning 2014 to 2023. The search keywords included (“agricultural greenhouse” or “greenhouse”) and (“control algorithm” or “control strategy”) and (“crop growth model” or “greenhouse microclimate model”). The search results showed a gradual increase in research in the field of greenhouses in 2014, focusing mainly on improving productivity and conserving energy through greenhouse environment control. During the literature search, irrelevant publications were excluded, and repeat articles were removed. Finally, 107 relevant articles from the past decade were selected for detailed study.

**Figure 1:**
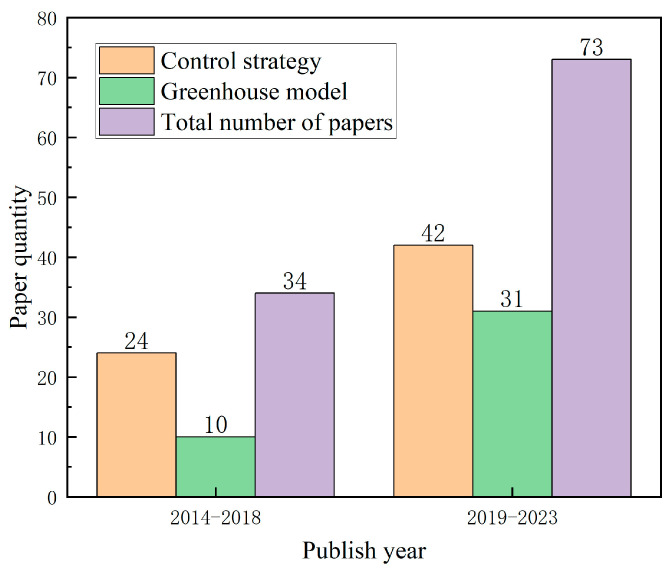
Statistics of papers published from 2014 to 2023.

Figure 1 shows the temporal distribution of academic journal articles and conference papers published from 2014 to 2023 according to the control strategy and the greenhouse model. Between 2014 and 2018, there was a comparatively lower volume of publications. However, in the subsequent period from 2019 to 2023, a noteworthy surge in research output emerged, particularly concerning control strategies and greenhouse models. This indicates an escalating involvement of scholars in the research of greenhouse control strategies and models.

## 3. Research on Control Strategy

Greenhouses provide a conducive growth environment for plants, whether in cold winters or hot summers. Their use enables plant growth in non-traditional growing seasons while continuously mitigating external environmental influences on plant growth. Energy conversion in greenhouses is critical to plant growth. By optimizing the control strategies of heating, cooling, lighting, and other systems, optimizing the energy conversion process of greenhouses not only promotes plant growth, but also enhances the efficiency of energy utilization and reduces production costs [37].

### 3.1. Structure Control

Due to the complexity and variability of greenhouse environments, effective control over them is of great significance. Altering the greenhouse environment through internal structural control is paramount. Current structural control mainly includes shade system control, ventilation system control, refrigeration system control, and heating system control.

The control of greenhouse structures mainly involves adjustments via controllers to achieve energy-saving objectives. Li et al. [38] investigated the performance of a blind-type shading regulator that automatically rotates semi-transparent photovoltaic (PV) blades installed on the greenhouse roof in response to sunlight variation. The results showed that the system was capable of generating 13 kWh m^−2^ yr^−1^ of surplus electricity on sunny days and partially met the electricity demand of the greenhouse on cloudy days. Xiao et al. [39] proposed an earth–air heat exchanger (EAHE) based on the characteristics of greenhouse structures. They calculated the heat exchange capacity of EAHE based on greenhouse heating and cooling loads, determining pipe length, diameter, air velocity, and burial depth by integrating heat exchange efficiency and the number of transfer units. Finally, they optimized the last parameters considering energy loss and construction costs. The results showed that this innovative structure had a significant energy-saving effect while meeting the internal temperature requirements of the greenhouse. Gutierrez-Arias et al. [40] devised an opening and closing control strategy for side ventilation windows, constructing a solving algorithm in MATLAB 2019a to achieve dynamic simulation of crop–microclimate interactions. The results illustrated that adjusting the opening and closing of these side ventilation windows effectively regulated the internal greenhouse CO_2_ concentration, temperature, and humidity conditions. Xu et al. [6] proposed a novel water circulation solar collector release system designed for indoor use. This collector worked as a heating radiator during nighttime, and achieved an average daily heat collection rate of 72.1%. Compared with other similar products, this collector had a larger effective area, which improved thermal efficiency and energy-saving performance. In summary, most of the current research on greenhouse structural control focuses only on individual systems in lighting, ventilation, cooling, and heating systems, and research on multi-system coupling is the key to solving the problem.

### 3.2. Parameter Control

Control of greenhouse environmental parameters relies on controllers for direct adjustments within the greenhouse environment, which is a key factor in plant growth. The primary environmental parameters of greenhouses include temperature, humidity, lighting, CO_2_ concentration, and wind speed [41]. Nowadays, in parameter control studies, a great number of scholars focus on researching temperature and humidity, while some researchers also study other parameters [42].

Greenhouse environmental parameters can be monitored and controlled through automated systems, using computers and controllers to achieve real-time responses to environmental changes. This ensures optimal growing conditions for crops in a greenhouse environment. Xiao et al. [8] used a greenhouse structural model, employing the fuzzy active disturbance rejection control technique to regulate the temperature inside the greenhouse. Revathi [43] applied the particle swarm optimization (PSO) algorithm for basic fuzzy control and greenhouse temperature fuzzy control. Chen [44] proposed an adaptive model predictive control method incorporating the feedback linearization technique to regulate the temperature in the greenhouse. Liang [45] used model predictive control to adjust the greenhouse temperature by changing the heater and ventilation windows. Montoya-Rios [46] proposed a control strategy using a natural ventilation system to regulate daytime greenhouse temperatures. Timmermans [47] suggested using advanced optical materials for lighting control. Tang [48] introduced an intelligent lighting system for greenhouse plants, providing intelligent supplementation in case of insufficient light. Selmani [49] used a solar photovoltaic water pumping system for greenhouse irrigation, which changed the humidity in the greenhouse. Sanchez-Molina [50] proposed a scheme for storing CO_2_ from flue gases, collecting CO_2_ to improve crop photosynthesis rates by supplying it to the greenhouse. Controlling greenhouse environmental parameters can significantly improve the plant growth environment. Future control methods may integrate multiple parameters and use altered control algorithms to achieve precise control of complex and dynamic greenhouse environments.

### 3.3. Control Algorithms

A greenhouse environment control system is a nonlinear, multi-input, and multi-output system. Greenhouse control mainly involves the collection of greenhouse environment and crop growth data via sensors. Afterwards, these data are utilized to formulate control rules, which are transmitted to the controller to achieve coordination between different control parts. Through scientific optimization and energy-saving operations, this careful arrangement promotes changes in greenhouse environmental conditions, ultimately promoting crop growth. Many control algorithms have been applied to the control of greenhouse systems: for instance, Proportional–Integral–Derivative (PID) control, fuzzy control, feedback control, adaptive control, model predictive control (MPC), and neural network control. These control algorithms will be discussed in detail.

#### 3.3.1. PID Control

**Figure 2:**
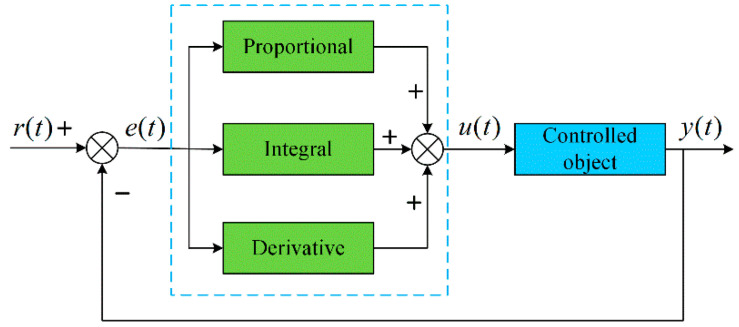
Schematic diagram of PID control principle.

The control of greenhouse environments is a typical control process. PID is one of the earliest and traditionally developed control strategies in agriculture [1]. Through the function operation of P, I, and D, the final output control instructions are used to control the greenhouse environment to meet the growth needs of crops. The traditional PID control principle is shown in Figure 2. Conventional PID control can only change the single factor of greenhouse environments in the control. However, PID control is difficult to simultaneously control multiple factors. Hu [51] proposed an economically tuned method for multiple PID controllers based on a non-dominated sorting genetic algorithm, combining various performance signs with production costs. The results showed that this controller achieved favorable control performance at relatively lower costs. Su [17] introduced a self-tuning parameter PID control method, validated through simulations using actual meteorological data to demonstrate its effectiveness and control performance. The results showed that the control system had a good control effect. Adesanya et al. [52] proposed a flexible PID controller that employs a deep reinforcement learning (DRL) algorithm to optimize its parameters, by tracking the setpoints and energy consumption of a greenhouse planted with tomatoes. The results showed that the proposed PID optimization parameters using the deep learning algorithm could reduce the energy consumption by 8.81% to 12.99%.

#### 3.3.2. Fuzzy Control

Fuzzy control is a nonlinear control method. Fuzzy control does not require an accurate mathematical model of the controlled object, and can control complex systems, such as multi-input, multi-output (MIMO), time-varying, and lag systems [12]. For the nonlinear and time-varying characteristics of the greenhouse internal environment, fuzzy control has a good control effect. In addressing the prolonged response time inherent in conventional greenhouse PID control, Cheng et al. [53] introduced an agricultural greenhouse temperature control model based on fuzzy PID. This model exhibited notable attributes, including reduced response time in temperature control and a consistent, stable temperature control effect. Its implementation demonstrated enhanced precision in regulating greenhouse temperatures.

**Figure 3:**
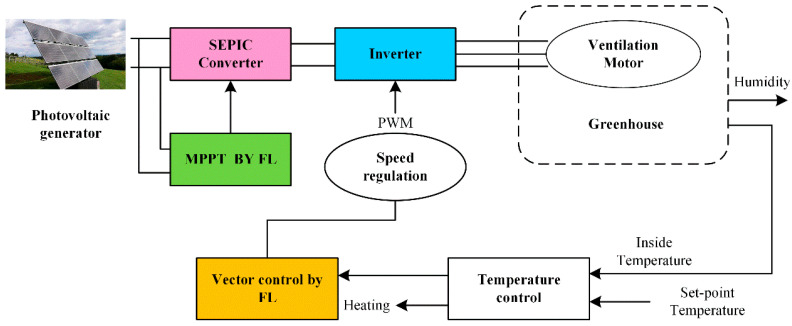
Schematic diagram of photovoltaic (PV)-based ventilation system.

To adjust the temperature and humidity in the greenhouse, Riahi [54] designed a multi-input and multi-output fuzzy controller, in which the ventilation, heating, and humidification functions in the greenhouse are controlled by the motor. The greenhouse climate is adjusted to the optimal state through a fuzzy control algorithm to control the actuator. In addition, the model of photovoltaic power generation is established, and the speed control of the asynchronous motor is carried out by vector control optimized by fuzzy logic to achieve an energy-saving purpose. The photovoltaic (PV)-based ventilation system is shown in Figure 3.

**Figure 4:**
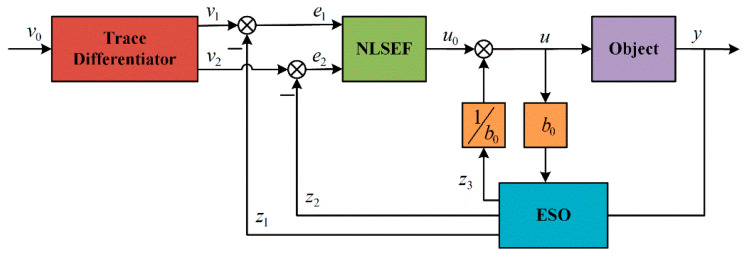
Schematic diagram of controller structure.

Xiao [8] introduced a fuzzy active disturbance rejection control method, which does not rely on an accurate mathematical model. This method displays commendable robustness and noise immunity. Through adjustment of the skylight’s opening and thermal air conditioning, it performs both greenhouse temperature regulation and energy conservation. The controller’s structure is depicted in Figure 4. Mac et al. [55] introduced a deep learning algorithm on the basis of the fuzzy control algorithm to form an upgraded Deep Convolutional Generative Adversarial Network (DCGAN). The results showed that the use of the proposed DCGAN improved the performance of the deep learning model for greenhouse plant monitoring and disease detection.

#### 3.3.3. Feedback Control

Regarding control system design, feedback control is a commonly used method that shows effective control performance in greenhouse applications [56]. Feedback control requires a precisely defined model of the controlled system and a well-crafted control algorithm [57].

Hoyo et al. [42] used a control strategy combining feedback linearization techniques and quantitative feedback theory (QFT) to control the indoor temperature of greenhouses. The feedback linearization control strategy was designed based on the nonlinear model of the greenhouse, and the PI controller was designed using QFT. The results showed that the control system had satisfactory control performance under the condition of disturbance or change in working position. To improve the performance of the cantilever sprayer in the greenhouse, Fu et al. [58] developed an automatic control technology based on speed feedback. It uses an auxiliary anti-drift system of wind curtain-type airflow and a variable spray control system for adaptive fertilization and environmental monitoring. The system was able to control the speed error within 3%. The airflow improved penetration spray, reduced the droplets, and improved the use rate.

However, although feedback control has better accuracy, stability, and robustness, feedback control requires accurate models and sensors with high accuracy, and the demands for control algorithms are also relatively high.

#### 3.3.4. Adaptive Control

Adaptive control systems have the capacity to automatically adjust themselves to account for changes in system parameters and external disturbances. They show exceptional performance when confronted with model confusions and variations in system parameters by displaying strong control performance in greenhouse applications [59].

Su [17] presented a parameter self-tuning PID control method. This approach involves the early conversion of internal temperature, humidity, and CO_2_ concentration into four comparable unit outputs. To ensure that the desired control performance was obtained, the Levenberg–Marquardt (LM) algorithm was used to tune the PID parameters.

Through the system identification toolbox, Wang et al. [60] identified the assumed parameter model and parameter state of the temperature in the greenhouse. The model transfer function was established, and the category of the model was determined. Finally, the system structure of the adaptive control was obtained, and the adaptive control of the system was realized. Wang et al. [61] proposed an adaptive fuzzy control algorithm for greenhouse temperature control. The adaptive control algorithm can select the appropriate time to open the rolling curtain according to the place of greenhouse. Boughamsa [62] considered the use of an adaptive T-S fuzzy model to predict future greenhouse environmental conditions in a control scheme, and the use of heating and ventilating to adjust the temperature and humidity inside the greenhouse. To accurately control the temperature inside the greenhouse, Chen et al. [44] proposed an adaptive model predictive control method combined with feedback linearization technology, and the control ability was significantly improved. Su et al. [4] proposed an indirect adaptive fuzzy control method for a class of MIMO nonlinear systems with unknown dynamics and actuator saturation for greenhouse climate control issues. This method efficiently tracks the temperature and humidity within the greenhouse while preserving control.

#### 3.3.5. Model Predictive Control (MPC)

An advanced control technology called model predictive control (MPC) was used for controlling dynamic systems in order to meet predetermined performance goals [63]. The basic principle of MPC is to forecast a system’s future behavior using a mathematical model and then produce control methods based on these forecasts [64].

**Figure 5:**
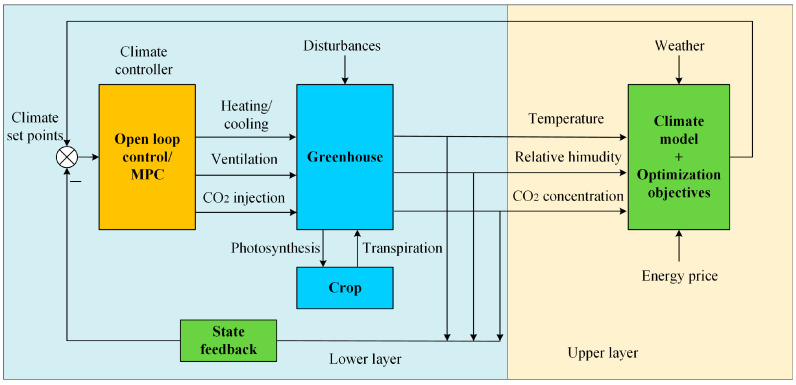
Schematic diagram of the structure of the greenhouse climate hierarchical control system.

Achour et al. [65] used MPC to propose a novel control strategy for the optimal operation of a microgrid powered greenhouse, which can maintain the optimal greenhouse microclimate, and can manage irrigation, lighting, ventilation, and dehumidification, and accomplish the goal of energy conservation. Bersani et al. [66] adopted the MPC method to keep the greenhouse’s temperature at its ideal level while consuming the least amount of energy. Jung [5] proposed an MPC-based greenhouse temperature optimization ventilation system with excellent control performance, which drives the operation of several interior windows of the greenhouse by forecasting internal temperature changes. Lin et al. [67] proposed a hierarchical control strategy for the Venlo greenhouse system. The upper layer sets different optimization learning objectives (different strategies), and the lower layer uses MPC to track the trajectory obtained from the upper layer, and the control performance of the MPC controller and the open-loop controller is compared. The results showed that the MPC had a better prediction accuracy. The greenhouse climate hierarchical control architecture is shown in Figure 5.

Aiming at the confusion in the greenhouse system, Mahmood et al. [68] proposed a data-driven robust model predictive control, which can be used for greenhouse temperature control and energy use evaluation. Compared with the basic model predictive control strategy, the robust model predictive control strategy reduced the energy consumption by 9.67% and 23.61% in winter and summer, respectively. Faced with the problem of excessive water use caused by traditional irrigation practices, Caceres et al. [69] proposed a periodic model predictive control structures for on–off irrigation. Under the conditions of both stable and unstable greenhouse environments, the controller had a good control effect.

#### 3.3.6. Neural Network Control

Neural network control is a machine learning model based on artificial neural networks, with multi-layer network parameters as the structure and most data as training samples [70]. The changing temperature and humidity data in the greenhouse have the characteristics of time series, which is consistent with the research of neural network time series [71]. The neural network model has strong self-organization, self-learning, and self-adaptive ability. The neural network model can not only predict the change state of indoor environmental factors, but also carry out the best control through prediction [72].

**Figure 6:**
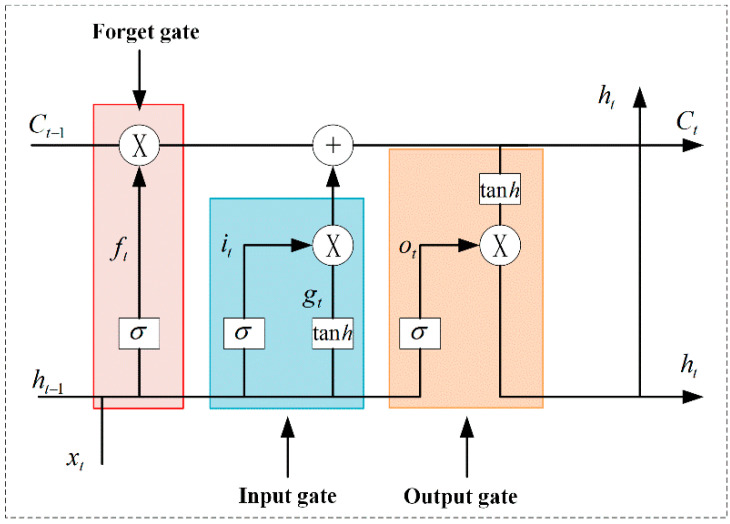
Schematic diagram of the LSTM neural network process.

Castaneda-Miranda et al. [73] used a multi-layer perceptron artificial neural network (ANN) to prevent frost in intelligent greenhouses. The system’s parameters include wind speed, the relative humidity of the outdoor air, total solar radiation flux, and the relative humidity of the inside air. Its temperature forecast accuracy can approach 95%. Fourati [74] used an Elman neural network to model the greenhouse environment to achieve multi-neural control of the greenhouse and improve the greenhouse environment climate. Jung et al. [75] designed a data-based tomato greenhouse evapotranspiration (ET) and humidity deep learning model for the issue of crop humidity change and crop transpiration prediction in greenhouses. The test dataset was used for humidity prediction. The prediction results were compared with the prediction results of the traditional Recurrent Neural Network–Long Short-Term Memory (RNN-LSTM) model, and the experiments showed that the model had better prediction performance. The Long Short-Term Memory (LSTM) neural network is shown in Figure 6. Liu et al. [76] proposed the Google Cloud Platform–Long Short-Term Memory (GCP-LSTM) model for greenhouse climate prediction, which can reliably forecast greenhouse climate. Mounir et al. [77] predicted the ventilation volume in the greenhouse through the RNN-LSTM model, and guided the opening degree of the vent according to the size of the air volume. Wu et al. [78] used a neural network to compensate the error of the temperature and humidity sensor, and the detection accuracy of the sensor was improved. The particle swarm optimization (PSO), Back Propagation (BP) neural network, and PID control algorithm were combined to construct the PSO-BP-PID control algorithm. The algorithm was used to control the temperature and humidity, which improved the response speed of the greenhouse system and reduced the overshoot. It was able to control the temperature and humidity in the greenhouse more accurately. Belhaj [79] introduced a deep learning algorithm on an Elman neural network to form a deep multi-layer perceptron (MLP) neural network to control the greenhouse internal climate.

#### 3.3.7. Hybrid Control

Hybrid control is an advanced control strategy, which combines different types of control methods to better cope with the diversity, nonlinearity, and uncertainty of the system [80]. Among the current control methods, each control method has its own advantages. Hybrid control is capable of capitalizing on the advantages of different control methods to achieve economical, convenient, and accurate control [81].

**Figure 7:**
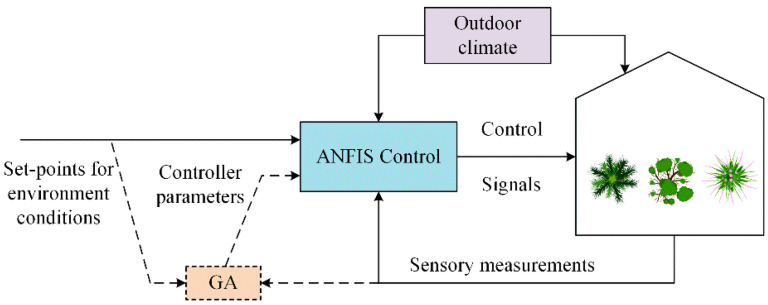
Schematic diagram of control structure by using GA to tune ANFIS parameters.

Castaneda-Miranda [82] introduced a defrosting control system, which was realized by integrating the Internet of Things (IoT) and hybrid AI methods in the greenhouse environment. The system employs an artificial neural network for greenhouse temperature prediction, achieving a remarkable accuracy of up to 95%. Afterwards, fuzzy control is employed to regulate the temperature based on the presence of frost in the greenhouse, and the purpose of defrost is effectively realized. Ding [83] proposed a monitoring system based on a Programmable Logic Controller (PLC) and Supervisory Control and Data Acquisition (SCADA); the system, combined with an artificial neural network, was able to predict the environmental parameters for Dendrobium officinale growth and achieve real-time monitoring and intelligent control. Mohamed [84] proposed an Adaptive Neuro-Fuzzy Inference System (ANFIS), combined with genetic algorithm (GA) to adjust the controller. According to the set value and the changing outdoor climate conditions, the temperature and humidity of the greenhouse system were adjusted to adapt to the changing climate conditions in the greenhouse. The control structure is shown in Figure 7. To solve the issue of switching between two different heating systems in the greenhouse to control the nighttime temperature, Montoya [85] designed a hybrid model-based predictive controller. The system dynamics were represented by a hybrid model. The model predictive hybrid controller was used to adjust the inner temperature at night, and the optimal control signal was calculated based on the power consumption and commutation minimization. Although hybrid control incorporates the advantages of various control algorithms and has higher accuracy and anti-interference capabilities, the complexity of hybrid control is also higher than other control algorithms and is difficult to implement. Therefore, it is essential to take into account the advantages and disadvantages of existing control algorithms in the research of hybrid control and choose the optimal control algorithm as the basis of hybrid control according to the characteristics of the actual problem.

### 3.4. Discussion

Over the past decade, researchers have mainly focused on neural network control and model predictive control strategies in greenhouse control studies. Additionally, methods such as fuzzy control, adaptive control, and feedback control have also been extensively researched. Table 1 summarizes the advantages, disadvantages, and application scenarios of different control algorithms. In future control algorithms, neural network control is expected to gradually replace traditional methods. This is mainly due to the neural network’s ability to avoid complex mathematical models, requiring merely a portion of the data for learning, and its capability to achieve accurate prediction and controller adjustment. Table 2 outlines the applications of neural network control and model predictive control (MPC) algorithms in greenhouse research. The control of greenhouse environmental parameters mainly includes temperature, humidity, CO_2_ concentration, light, and wind speed. The environmental parameters of greenhouses exhibit strong coupling characteristics. In future research, multi-parameter coupling control is expected to become a new research focus.

## 4. Research on Greenhouse Model

Greenhouse models mainly include greenhouse microclimate models and crop growth models, which simulate various environmental parameters and crop growth in greenhouses [86]. They contribute to a better understanding of the response of agricultural systems to climatic conditions and the potential impact of climate change on agricultural production.

### 4.1. Greenhouse Microclimate Models

Greenhouse microclimate models mainly simulate environmental conditions in the greenhouse, including factors such as temperature, humidity, CO_2_ concentration, and lighting [87]. These models can better control the growing environment of greenhouse crops and help provide optimal growing conditions. This section describes the research tools commonly used to construct greenhouse microclimate models, including artificial neural network (ANN) models, computational fluid dynamics (CFD), Comsol Multiphysics 6.1 (COMSOL6.1), Matrix Laboratory 2022a (MATLAB2022a), C++, etc. Table 3 summarizes the research tools used to construct greenhouse microclimate models.

#### 4.1.1. Artificial Neural Network

In recent years, research on greenhouse microclimate models has extensively employed ANN technology. ANN models forecast the microclimate in greenhouses, provide future greenhouse climate data, and guide greenhouse control. Because of their precise predictive abilities, ANNs have gained widespread acclaim among research scholars.

ANN is a key tool for data analysis and forecasting in greenhouse agriculture, using strong learning capabilities and the ability to identify complexities. They contribute to the effective management and optimization of agricultural production. Jung [97] developed ANN, Nonlinear Autoregressive with Exogenous Inputs (NARX), and RNN-LSTM models to forecast temperature, humidity, and CO_2_ concentration for crop growth in greenhouses. The results demonstrated that the RNN-LSTM prediction model exhibited the highest accuracy within a 30 min timeframe. Wang et al. [90] introduced a dynamic BP algorithm model based on recursive neural networks, and compared it with RNN neural networks. The findings showed the RNN-BP prediction model exhibited superior predictive performance for temperature and humidity in the greenhouse. Gharghory et al. [99] proposed a recursive neural network prediction model based on Long Short-Term Memory (LSTM), and compared its predictive values with the traditional BP-ANN prediction model. The variance coefficient R^2^ of the LSTM prediction model exceeded 0.997, indicating superior predictive accuracy compared to the traditional BP-ANN model. Zhao et al. [102] introduced a climate prediction model based on LSTM to forecast temperature, humidity, and CO_2_ concentration in greenhouses. Their predictions, conducted across various greenhouse settings, consistently demonstrated favorable forecast accuracy. Yang et al. [103] developed a Feedforward Attention Mechanism–LSTM (FAM-LSTM) model to forecast temperature and humidity in solar greenhouses. This model comprehensively incorporates both internal and external environmental factors that affect crop growth. Comparative analysis with other prediction models showed that FAM-LSTM had excellent predictive accuracy. Liu [101] used BP neural networks and stepwise regression to establish microclimate prediction models for different seasons in mushroom greenhouses. Through the comparative analysis of the two prediction algorithms, it was discovered that the prediction accuracy of BP neural network model was significantly higher than that of stepwise regression model.

#### 4.1.2. Computational Fluid Dynamics

CFD is a simulation and modeling technique known for its high accuracy in predicting fluid flow inside or outside structures and equipment [104]. In simulating greenhouse microclimates, CFD is mainly used to model the dynamic fluid conditions in the greenhouse, demonstrating good applicability.

CFD simulation techniques enable accurate modeling of environmental data in greenhouses, to better understand and control the greenhouse climate. This optimization improves crop growing conditions and increases production efficiency. In relevant research, Villagran [91] adopted CFD to simulate airflow and ventilation in three commercial greenhouses in the high Andean tropics region. They developed a transient CFD-2D model, which was solved using the finite volume method. The results showed that the model can effectively predict greenhouse temperature and wind speed. Bouhoun et al. [88] developed a two-dimensional transient CFD model considering the interaction between crops and the microclimate in the greenhouse. By analyzing moisture balance conditions under both enough and restricted water scenarios, the results indicated that the model could accurately predict transpiration, indoor temperature, and humidity under these two moisture conditions. Saberian [94] established a three-dimensional CFD model to simulate airflow and heat transfer in greenhouse. Considering solar radiation loads and local wind directions, the model predicted daily microclimate variations in the greenhouse. It accurately forecasted changes in solar heat loads, identified periods when indoor temperatures exceeded ambient temperatures, and proposed cooling strategies using fan-assisted natural ventilation during specific time frames for cold-climate cultivation regions. Ernesto [100] used CFD simulations to forecast temperature variations in greenhouses with supplementary heating equipment. The study estimated the energy consumption required to maintain the greenhouse climate. Chalill [100] conducted full-scale simulations of environmental parameters for crops in the greenhouse, predicting the spatial distribution of crop growth parameters. The simulated values closely aligned with experimental data. Li [105] established a greenhouse microclimate model by introducing a hybrid computational fluid dynamics–evolutionary algorithm method. This model incorporated the influence of environmental factors on crop growth, deriving optimal crop growth environment parameters. The accuracy of the CFD simulation was verified by comparing the predicted results with the experimental results. Finally, Pakari [92] employed CFD simulations to assess the ventilation efficacy of greenhouses equipped with wind towers. By analyzing airflow exchange rates and distributions under varying wind speeds and incident angle conditions, the results indicated that the simulated wind speeds aligned with experimental measurements within a 12% margin.

#### 4.1.3. Other Simulation Software

Research on simulating greenhouse microclimates mainly focuses on CFD and ANN. Additionally, some researchers use software such as COMSOL and MATLAB, while previous studies have also used programming languages such as C++ and Fortran to construct models.

Ma [93] used COMSOL to establish a greenhouse microclimate model to predict temperatures and light radiation at different locations and time intervals. Comparing the predicted values with actual ones, the simulation accurately forecasted the environment within the greenhouse. Sciuto [89] used COMSOL to construct a model to study the efficiency of greenhouse Heating Ventilation and Air Conditioning (HVAC) systems. The simulation was carried out under different climatic conditions, and the results showed that the simulated values of temperature and wind speed were consistent with the experimental values. Yau [95] designed a dynamic model for the internal air temperature of a solar greenhouse in the MATLAB/Simulink environment. This model incorporated factors such as solar radiation and surrounding temperature to formulate an energy balance equation. The simulation precisely predicted the change in temperature inside the greenhouse. Katzin [106] introduced a supplementary lighting model for greenhouses using MATLAB. This model analyzed the impact of supplementary lighting on greenhouse climates and crops, and compared differences between high-pressure sodium lamps and light-emitting diode lamps. It also predicted greenhouse heating requirements. The results demonstrated a prediction error of 8–51 Wm^−2^ for the greenhouse heating demand. Ali [96] developed a dynamic model for greenhouse thermal behavior using MATLAB/SIMULINK R2017b. This model simulated the variations in greenhouse air temperature considering both transparent and insulated greenhouses. By comparing experimental data with simulation values, the results indicated a close match between the simulated and actual values.

### 4.2. Crop Growth Model

**Figure 8:**
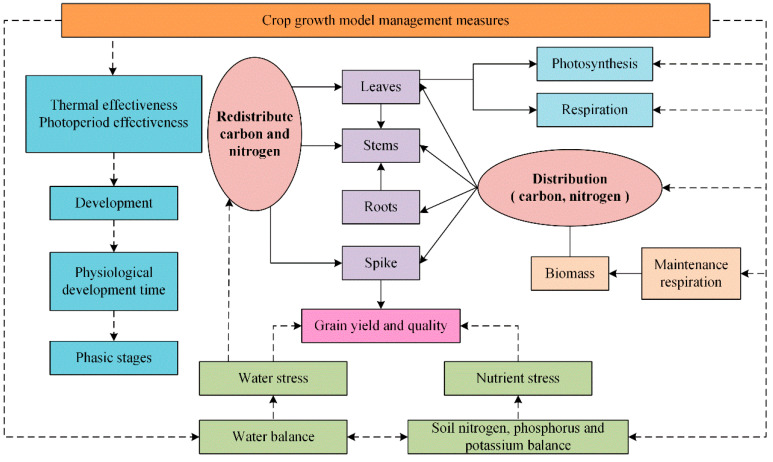
Structure flow chart of crop growth model.

Crop growth models are mainly based on the developmental patterns and productivity formation of crops to establish mechanistic models of crop growth. These models simulate the growth and development of various plant organs (leaves, stems, roots, etc.), photosynthesis, and nutrient transport. By promoting improved regulation of crop growth, they can predict the processes of crop growth, development, yield, and quality formation. The fundamental model of crop growth is illustrated in Figure 8.

Researchers have employed various crop growth models to simulate and predict the growth conditions of different crops under diverse environmental settings. These models are rooted in different theories and methods, which are beneficial for simulating and predicting crop growth conditions. Huang [107] used the WOFOST crop growth model to assess the yield of winter wheat. They compared three leaf area index (LAI) datasets with different temporal and spatial resolutions and found that assimilating the Kalman filter algorithm (KF) and LAI into the WOFOST model can significantly improve the accuracy of crop yield predictions. Whish [35] designed a crop growth model integrated with the Agricultural Production Systems simulator (APSIM) in DYMEX. They further studied biological constraints during crop growth by advancing the development of the APSIM model and investigating how fungal populations reduce the crop leaf area. Alderman [34] used the Cropgro crop growth model to simulate the growth and development of Pigeonpea. They adjusted the parameters of the growth model to suit Pigeonpea growth and validated it using datasets. The study demonstrated that Cropgro can effectively simulate the growth and development process of Pigeonpea. Ma [108] combined the parameter estimation software PEST V3 with the DSSAT V4.7 crop growth model. They adopted the newly developed DSSAT-PEST package to automatically optimize crop parameters and compared three parameter optimization methods. The results indicated that DSSAT-PEST provided an accurate and effective method for estimating crop genetic parameters, which significantly improved efficiency and accuracy. Boote [109] used the Cropgro crop growth model to simulate carinata growth. Through the analysis of growth data from three sites and conducting sensitivity analyses, they developed the Cropgro–carinata crop growth model. This model accurately simulated climate parameters, soil characteristics, and growth traits during crop growth. Compared to the experimental results, the improved model demonstrated strong abilities in simulating the dynamic growth of crops. To improve resource use in crops, Wu [110] developed a cross-scale model by coupling photosynthesis and crop growth models to provide clearer insights into the impact of photosynthesis on the environment and crop growth. This model provides technical support for promoting crop growth. Graefe et al. [111] proposed a novel leaf canopy photosynthesis model focusing on the growth mechanism of crop leaves. This model can predict the light adaptation capability of tomato crops over a two-month period. Nomura et al. [112] proposed a method integrating canopy photosynthesis model and image analysis to estimate greenhouse air quality. By focusing on the crop canopy region, they constructed an energy balance equation including single-leaf photosynthesis, stomatal conductance, and mass transfer. This method allows the long-term estimation of spinach photosynthesis rates and transpiration rates under different calcium levels. Chen et al. [113] developed a maize growth model to investigate crop growth dynamics under different soil moisture and coverage conditions. The model simulates crop growth rates, crop yield, and water use efficiency during both day and night with high simulation accuracy.

### 4.3. Discussion

Greenhouse microclimate models typically focus on simulating internal environmental parameters, mainly using ANN, CFD, COMSOL, and similar software for model construction. These models mainly focus on simulating temperature, humidity, and illumination in the greenhouse. However, there is a lack of simulation about CO_2_ concentration and crop transpiration rates. A comprehensive greenhouse microclimate model should include the simulation of all environmental parameters in the greenhouse. Crop growth models simulate crop growth, dynamic environments, and the growth and development of different plant organs. They also provide good predictive accuracy for yield and quality. However, current crop growth models still rely on empirical data, which affects their universality [114,115]. Some crop growth models have integrated pest and disease models, mechanistically explaining the impact of harmful organisms on crop growth. Nevertheless, research on economic and sociocultural aspects within these models is still insufficient. In addition, the coupling of greenhouse microclimate models and crop growth models is understudied, as the environmental parameters of greenhouse microclimate models can have an effect on crop growth (e.g., the effects of temperature, humidity, and CO_2_ concentration on transpiration and photosynthesis), and similarly, the crop growth process can change the environmental parameters of greenhouse microclimate. Therefore, future research should focus on integrating greenhouse microclimate models and crop growth models to form more comprehensive greenhouse microclimate models that take into account economic and sociocultural factors to create more comprehensive, harmonized, and universally applicable crop growth models.

## 5. Conclusions and Foresight

This review includes studies on greenhouse control strategies, greenhouse microclimate models, and crop growth models over the past decade. The summary is as follows:

1. Regarding greenhouse control strategies, this paper extensively studies greenhouse structural control, environmental parameter control, and control algorithms. In terms of greenhouse structural control, it mainly focuses on heating systems, ventilation systems, and cooling system control. In terms of parameter control, it develops from early temperature and humidity control to supplementary lighting and CO_2_ enrichment to improve photosynthetic efficiency of crops. In terms of greenhouse environmental control algorithms, the research on the greenhouse environment control algorithm indicates that PID control is the simplest and most widely used method among various control methods. However, with the increasing demand for greenhouse control, PID control is no longer sufficient. In recent years, neural network control and model predictive control have become a hot research topic, and a substantial number of studies have studied these two control methods.
2. In the greenhouse modeling, there are mainly two categories: the greenhouse microclimate model and the crop growth model. The greenhouse microclimate model involves the simulation of internal environmental parameters. There is a copious amount of literature that uses tools such as ANN and CFD to simulate the greenhouse microclimate. This modeling mainly includes the simulation of temperature, humidity, and light intensity in the greenhouse environment. On the other hand, the crop growth model mainly focuses on simulating the growth mechanisms of crops, investigating their growth dynamics, yield, and related aspects. Among these models, the WOFOST model has had a significant impact and has been widely applied in global food production research. Currently, most crop growth models predominantly emphasize the growth of crops, and there is relatively limited research to understand the influence of pests and diseases on crop growth, as well as the impact of socioeconomic factors.

**Figure 9:**
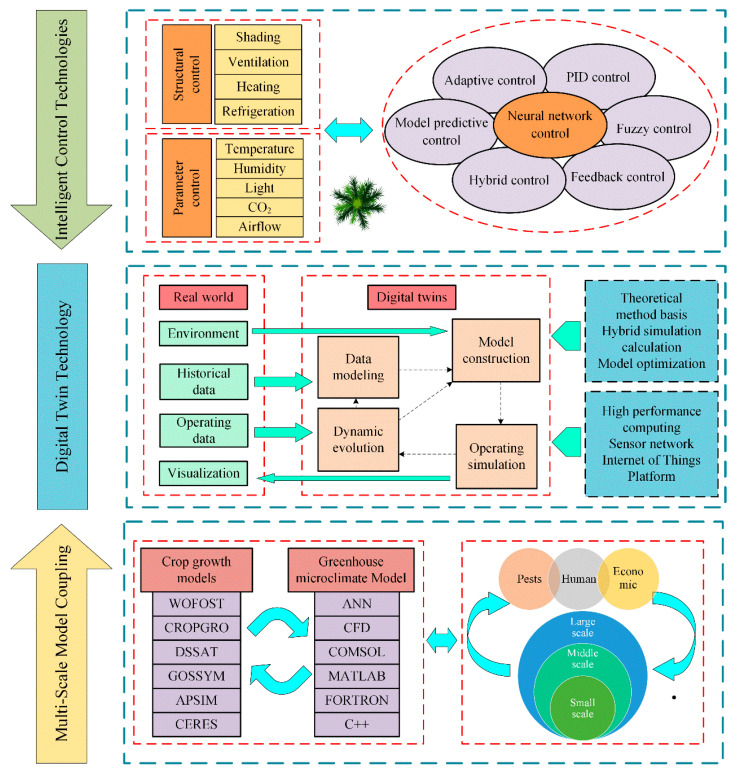
Schematic diagram of future research direction.

With the continuous progress of science and technology, future greenhouse control strategies and models are expected to exhibit a trend towards the integration of multiple models. Figure 9 provides an outline of the key areas for future research.

3. Coupling of Intelligent Control Technologies

With the rapid development of artificial intelligence and big data, the future will witness the integration of ANN with various optimization algorithms. This coupling will improve the prediction accuracy of ANN and use optimization algorithms to seek the best solution of system control parameters and decision variables. The control precision of greenhouse can be improved significantly by combining different control methods and maximizing the utilization of their respective advantages.

4. Multi-Scale Model Coupling

In the future, crop growth models will surpass singular crop growth and yield simulations by integrating with greenhouse microclimate models. Additionally, incorporating remote sensing techniques into greenhouse models, as well as the consideration of factors such as pests, diseases, socioeconomic aspects, and human-related factors, will facilitate multi-scale integration of different models. These scales encompass global, regional, and local perspectives, fundamentally improving the universality and accuracy of the models.

5. Digital Twin Technology

The future application of digital twin technology in the greenhouse field is anticipated to expand significantly. This technology utilizes mathematical modeling and simulation to analyze greenhouse systems to improve agricultural production, resource sustainability, and energy conservation. Digital twin technology not only simulates greenhouse environments but also models crop growth. It optimizes energy utilization in greenhouses and improves greenhouse structures. In addition, it shows great promise for training and educating greenhouse management personnel. The prospects for digital twin technology in the greenhouse sector are exceedingly promising.

## Figures and Tables

**Table 1:** Advantages and disadvantages of different control algorithms and application scenarios.

S/N	Control Algorithms	Advantage	Disadvantage	Application Scenarios
1	PID control	1. Simple controller structure	1. Difficult to control multiple variables at the same time	1. Individual parameter control 2. Primary greenhouse control
2	Fuzzy control	1. No need for accurate modeling	1. Poor real-time performance 2. Constrained by the rule base	1. Multivariate greenhouse system 2. Control of nonlinear systems
3	Feedback control	1. Highly accurate 2. Predictable response behavior	1. High sensor accuracy requirements	1. Scenarios for precise environmental control 2. Real-time control requirements
4	Adaptive control	1. High system adaptive control capability 2. Easy self-optimization	1. Initial-phase system instability 2. Difficulty in system development and maintenance	1. Occasions for self-adjustment of control strategies in response to environmental changes
5	Model predictive control	1. Predicting the future dynamic behavior of the system 2. Multi-constraint optimization capability	1. Computational complexity 2. High model dependency	1. Multivariate systems control 2. Complex system dynamics
6	Neural network control	1. Good self-learning skills 2. Ability to process time series data	1. High data demand 2. High training time and computational resource requirements	1. Application scenarios combining prediction and control 2. Systems requiring adaptive capabilities
7	Hybrid control	1. Integrating multiple control methods 2. High control accuracy and response speed	1. Complex design and commissioning 2. High real-time requirements 3. Difficult and costly to realize	1. Multivariable and uncertain systems 2. Systems requiring adaptive capabilities 3. Scenarios combining prediction and control

**Table 2:** Research overview of neural network control and MPC.

S/N	Control Algorithm	Controlled Parameter	Result of Study	Year and Reference
1	ANN	Frost	ANN was used for greenhouse control, and the accuracy of temperature prediction was as high as 95%.	2017 [73]
2	MPC	Temperature, humidity, light	The control was able to maintain the best greenhouse microclimate, and manage irrigation and lighting.	2020 [65]
3	MPC	Temperature	The greenhouse temperature-optimized ventilation system was able to drive the opening and closing of indoor windows through temperature changes.	2020 [5]
4	MPC	Temperature, humidity, CO_2_ concentration	Using the hierarchical control strategy, the greenhouse environment was accurately predicted and controlled.	2020 [67]
5	MPC	Temperature	The temperature in the greenhouse was maintained by consuming the least amount of energy.	2021 [66]
6	RNN-LSTM	Humidity	The data-based tomato greenhouse evapotranspiration (ET) and humidity deep learning model predicted the relative humidity of the greenhouse, and the prediction accuracy was significantly better than the traditional RNN-LSTM.	2022 [75]
7	GCP-LSTM	Temperature, humidity, light, CO_2_ concentration soil temperature, soil humidity.	The GCP_LSTM prediction model was tested on three different crops and compared with other prediction models; GCP_LSTM had a better prediction accuracy.	2022 [76]
8	Robust-MPC	Temperature, energy	Compared with the basic model predictive control strategy, the robust model predictive control strategy reduced the energy consumption by 9.67% and 23.61% in winter and summer, respectively.	2023 [68]
9	MPC	Humidity	A stable control effect was achieved by using MPC to control the irrigation system.	2023 [69]
10	RNN-LSTM	Ventilation volume	The opening degree of the vent was guided according to the size of the air volume.	2023 [77]
11	PSO-BP-PID	Temperature, humidity	The detection accuracy of the sensor was improved, the reaction speed of the control system was controlled, and the overshoot was reduced.	2023 [78]

**Table 3:** Overview of greenhouse microclimate modeling research tools.

S/N	Simulation Tool	Simulation Parameters	Result	Reference	Year
1	CFD	Transpiration, temperature, humidity	The CFD model was used to predict the transpiration, indoor temperature, and indoor humidity in the greenhouse.	[88]	2018
2	COMSOL	Temperature, wind speed	Under different climatic conditions, the simulated values of temperature and wind speed in the greenhouse were consistent with the experimental values.	[89]	2018
3	RNN-BP	Temperature, humidity	Compared with the greenhouse prediction ability of RNN, RNN-BP had a higher accuracy in greenhouse temperature and humidity prediction.	[90]	2018
4	CFD	Temperature, wind speed	The CFD-2D model was established, which can predict the temperature and wind speed in the greenhouse.	[91]	2019
5	CFD	Air current	The ventilation rate and airflow distribution in the greenhouse with different wind speeds and different incidence angles were analyzed. The error between the simulated value and the experimental values was less than 12%.	[92]	2019
6	COMSOL	Temperature, light radiation	Using the model to predict the greenhouse environment for 24 h and 7 days, the temperature and light radiation at different positions and different times in the greenhouse were accurately predicted.	[93]	2019
7	CFD	Light, temperature	A higher greenhouse temperature than outdoor temperature during the day was simulated, and a fan was used to assist natural ventilation for cooling.	[94]	2019
8	MATLAB	Temperature	A dynamic model of air temperature in greenhouse was established, which accurately predicted the change in temperature in the greenhouse.	[95]	2020
9	MATLAB	Temperature	The transparent greenhouse and heat insulation greenhouse were simulated, and the simulated values were in good agreement with the actual values.	[96]	2020
10	RNN-LSTM	Temperature, humidity, CO_2_ concentration	The RNN-LSTM model had the highest prediction accuracy for temperature, humidity, and CO_2_ concentration at 30 min.	[97]	2020
11	CFD	Temperature, heater position, heater direction	Auxiliary equipment was installed to heat the greenhouse, and the influence of the heater position and outlet direction on the greenhouse temperature was simulated.	[98]	2020
12	LSTM	Temperature, humidity	The prediction ability of the LSTM model was better than that of the RNN and BP-RNN models; the RMSE of the LSTM model for predicting temperature and humidity in the greenhouse was 0.16 and 0.62, respectively.	[99]	2020
13	CFD	Wind speed, temperature, humidity	The box-type evaporative cooler (BTEC) was used as the backbone of the HVAC system, and the environmental parameters in the greenhouse were monitored for 20 h. The results showed that the parameters in the greenhouse were within the required range.	[100]	2021
14	BP	Temperature	The BP neural network demonstrated high accuracy for greenhouse temperature prediction in different seasons.	[101]	2022
15	LSTM	Temperature, humidity, CO_2_ concentration	The model was transferred to other greenhouses, and demonstrated good prediction accuracy for indoor temperature, humidity, and CO_2_ concentration.	[102]	2022
16	LSTM	Temperature, humidity	The model predicted the temperature and humidity of the four time periods. The greenhouse prediction error of the four prediction layers was [−2.1,2.9] °C, and the humidity prediction error of the four prediction layers was [−6.9,4.4]%.	[103]	2023

## References

[1] Zhang S., Guo Y., Zhao H., Wang Y., Chow D., Fang Y. (2020). Methodologies of control strategies for improving energy efficiency in agricultural greenhouses. J. Clean. Prod..

[2] Iddio E., Wang L., Thomas Y., McMorrow G., Denzer A. (2020). Energy efficient operation and modeling for greenhouses: A literature review. Renew. Sustain. Energy Rev..

[3] van Beveren P.J.M., Bontsema J., van Straten G., van Henten E.J. (2015). Optimal control of greenhouse climate using minimal energy and grower defined bounds. Appl. Energy.

[4] Su Y., Xu L., Li D. (2016). Adaptive Fuzzy Control of a Class of MIMO Nonlinear System With Actuator Saturation for Greenhouse Climate Control Problem. IEEE Trans. Autom. Sci. Eng..

[5] Jung D.-H., Kim H.-J., Kim J.Y., Lee T.S., Park S.H. (2020). Model Predictive Control via Output Feedback Neural Network for Improved Multi-Window Greenhouse Ventilation Control. Sensors.

[6] Xu W., Song W., Ma C. (2020). Performance of a water-circulating solar heat collection and release system for greenhouse heating using an indoor collector constructed of hollow polycarbonate sheets. J. Clean. Prod..

[7] Sun X., Yang H., Liu Q.-F., Liu Y.-H. (2021). Event-triggered control for greenhouse temperature under natural ventilation based on computational fluid dynamics. Syst. Sci. Control Eng..

[8] Fei X., Xiao-Long W., Yong X. (2021). Development of Energy Saving and Rapid Temperature Control Technology for Intelligent Greenhouses. IEEE Access.

[9] Gerasimov D.N., Lyzlova M.V. (2014). Adaptive control of microclimate in greenhouses. J. Comput. Syst. Sci. Int..

[10] Liu R., Guzman J.L., Garcia-Manas F., Li M. (2023). Selective temperature and humidity control strategy for a chinese solar greenhouse with an event-based approach. Rev. Iberoam. Automática Informática Ind..

[11] Mishra K., Stanghellini C., Hemming S. (2023). Technology and Materials for Passive Manipulation of the Solar Spectrum in Greenhouses. Adv. Sustain. Syst..

[12] Chaudhary G., Kaur S., Mehta B., Tewani R. (2019). Observer based fuzzy and PID controlled smart greenhouse. J. Stat. Manag. Syst..

[13] Li Y., Ding Y., Li D., Miao Z. (2018). Automatic carbon dioxide enrichment strategies in the greenhouse: A review. Biosyst. Eng..

[14] Espinoza K., Valera D.L., Torres J.A., Lopez A., Molina-Aiz F.D. (2015). An Auto-Tuning PI Control System for an Open-Circuit Low-Speed Wind Tunnel Designed for Greenhouse Technology. Sensors.

[15] Santolini E., Pulvirenti B., Benni S., Barbaresi L., Torreggiani D., Tassinari P. (2018). Numerical study of wind-driven natural ventilation in a greenhouse with screens. Comput. Electron. Agric..

[16] Ferrante A., Mariani L. (2018). Agronomic Management for Enhancing Plant Tolerance to Abiotic Stresses: High and Low Values of Temperature, Light Intensity, and Relative Humidity. Horticulturae.

[17] Su Y., Yu Q., Zeng L. (2020). Parameter Self-Tuning PID Control for Greenhouse Climate Control Problem. IEEE Access.

[18] Yao J. Applications of Fuzzy Control in Greenhouse Intelligent Control System. Proceedings of the 1st International Conference on Information Sciences, Machinery, Materials and Energy (ICISMME).

[19] Mavkov B., Strecker T., Zecchin A.C., Cantoni M. (2022). Modeling and Control of Pipeline Networks Supplied by Automated Irrigation Channels. J. Irrig. Drain. Eng..

[20] Wang Y., Lu Y., Xiao R. (2021). Application of Nonlinear Adaptive Control in Temperature of Chinese Solar Greenhouses. Electronics.

[21] Lin D., Zhang L., Xia X. (2021). Model predictive control of a Venlo-type greenhouse system considering electrical energy, water and carbon dioxide consumption. Appl. Energy.

[22] Manonmani A., Thyagarajan T., Elango M., Sutha S. (2018). Modelling and control of greenhouse system using neural networks. Trans. Inst. Meas. Control.

[23] Escamilla-Garcia A., Soto-Zarazua G.M., Toledano-Ayala M., Rivas-Araiza E., Gastelum-Barrios A. (2020). Applications of Artificial Neural Networks in Greenhouse Technology and Overview for Smart Agriculture Development. Appl. Sci..

[24] Favela A., Capriles D. (2017). Hybrid System Control Using Discrete State Space Analysis. IEEE Lat. Am. Trans..

[25] Mirzamohammadi S., Jabarzadeh A., Shahrabi M.S. (2020). Long-term planning of supplying energy for greenhouses using renewable resources under uncertainty. J. Clean. Prod..

[26] Guo Y., Zhao H., Zhang S., Wang Y., Chow D. (2021). Modeling and optimization of environment in agricultural greenhouses for improving cleaner and sustainable crop production. J. Clean. Prod..

[27] Jiankun G., Yanfei L., Zengjin L., Xuewen G., Cundong X. (2019). Comparing the performance of greenhouse crop transpiration prediction models based on ANNs. J. Environ. Biol..

[28] Bazgaou A., Fatnassi H., Bouharroud R., Tiskatine R., Wifaya A., Demrati H., Bammou L., Aharoune A., Bouirden L. (2023). CFD Modeling of the Microclimate in a Greenhouse Using a Rock Bed Thermal Storage Heating System. Horticulturae.

[29] Cakir U., Sahin E. (2015). Using solar greenhouses in cold climates and evaluating optimum type according to sizing, position and location: A case study. Comput. Electron. Agric..

[30] Li Y., Zhou Q., Zhou J., Zhang G., Chen C., Wang J. (2014). Assimilating remote sensing information into a coupled hydrology-crop growth model to estimate regional maize yield in arid regions. Ecol. Model..

[31] Baishya A., Mishra A., Sengupta S. (2023). Modelling and Assessment of Climate Change Impact on Rainfed Rice Cultivation in a Sub-humid Subtropical Region. Agric. Res..

[32] Beegum S., Timlin D., Reddy K.R., Reddy V., Sun W., Wang Z., Fleisher D., Ray C. (2023). Improving the cotton simulation model, GOSSYM, for soil, photosynthesis, and transpiration processes. Sci. Rep..

[33] Zha H., Lu J., Li Y., Miao Y., Kusnierek K., Batchelor W.D. In-season calibration of the CERES-Rice model using proximal active canopy sensing data for yield prediction. Proceedings of the 13th European Conference on Precision Agriculture (ECPA).

[34] Alderman P.D., Boote K.J., Jones J.W., Bhatia V.S. (2015). Adapting the CSM-CROPGRO model for pigeonpea using sequential parameter estimation. Field Crops Res..

[35] Whish J.P.M., Herrmann N.I., White N.A., Moore A.D., Kriticos D.J. (2015). Integrating pest population models with biophysical crop models to better represent the farming system. Environ. Model. Softw..

[36] de Wit A., Boogaard H., Fumagalli D., Janssen S., Knapen R., van Kraalingen D., Supit I., van der Wijngaart R., van Diepen K. (2019). 25 years of the WOFOST cropping systems model. Agric. Syst..

[37] Zhang M., Yan T., Wang W., Jia X., Wang J., Klemes J.J. (2022). Energy-saving design and control strategy towards modern sustainable greenhouse: A review. Renew. Sustain. Energy Rev..

[38] Li Z., Yano A., Yoshioka H. (2020). Feasibility study of a blind-type photovoltaic roof-shade system designed for simultaneous production of crops and electricity in a greenhouse. Appl. Energy.

[39] Xiao J., Hu Y., Wang Q., Li J. (2023). Structural design method, validation, and performance analysis of an earth-air heat exchanger for greenhouses. Geothermics.

[40] Gutierrez-Arias E.M., Flores-Mena J.E., Pere-Osorio G., Morin-Castillo M.M., Pantle-Cuatle G., Cordova-Gutierrez E.J. (2021). Design and simulation of a control for the opening and closing of the side ventilation windows in a greenhouse. Rev. Mex. Fis..

[41] Chen W.-H., You F. (2022). Semiclosed Greenhouse Climate Control Under Uncertainty via Machine Learning and Data-Driven Robust Model Predictive Control. IEEE Trans. Control Syst. Technol..

[42] Hoyo A., Moreno J.C., Guzman J.L., Rodriguez F. (2019). Robust QFT-Based Feedback Linearization Controller of the Greenhouse Diurnal Temperature Using Natural Ventilation. IEEE Access.

[43] Revathi S., Sivakumaran N. Fuzzy Based Temperature Control of Greenhouse. Proceedings of the 4th IFAC Conference on Advances in Control and Optimization of Dynamical Systems (ACODS 2016).

[44] Chen L., Du S., Liang M., He Y. Adaptive Feedback Linearization-based Predictive Control for Greenhouse Temperature. Proceedings of the 6th International-Federation-of-Automatic-Control (IFAC) Conference on Bio-Robotics (BIOROBOTICS).

[45] Liang M.-H., Chen L.-J., He Y.-F., Du S.-F. Greenhouse temperature predictive control for energy saving using switch actuators. Proceedings of the 6th International-Federation-of-Automatic-Control (IFAC) Conference on Bio-Robotics (BIOROBOTICS).

[46] Montoya-Rios A.P., Garcia-Manas F., Guzman J.L., Rodriguez F. (2020). Simple Tuning Rules for Feedforward Compensators Applied to Greenhouse Daytime Temperature Control Using Natural Ventilation. Agronomy.

[47] Timmermans G.H., Hemming S., Baeza E., van Thoor E.A.J., Schenning A.P.H.J., Debije M.G. (2020). Advanced Optical Materials for Sunlight Control in Greenhouses. Adv. Opt. Mater..

[48] Tang Y., Jia M., Mei Y., Yu Y., Zhang J., Tang R., Song K. (2019). 3D intelligent supplement light illumination using hybrid sunlight and LED for greenhouse plants. Optik.

[49] Selmani A., Outanoute M., Oubehar H., Ed-Dahhak A., Lachhab A., Guerbaoui M., Bouchikhi B. (2019). An Embedded Solar-Powered Irrigation System Based on a Cascaded Fuzzy Logic Controller. Asian J. Control.

[50] Sanchez-Molina J.A., Reinoso J.V., Acien F.G., Rodriguez F., Lopez J.C. (2014). Development of a biomass-based system for nocturnal temperature and diurnal CO_2_ concentration control in greenhouses. Biomass Bioenergy.

[51] Hu H.G., Xu L.H., Goodman E.D., Zeng S.W. (2014). NSGA-II-based nonlinear PID controller tuning of greenhouse climate for reducing costs and improving performances. Neural Comput. Appl..

[52] Adesanya M.A., Obasekore H., Rabiu A., Na W.-H., Ogunlowo Q.O., Akpenpuun T.D., Kim M.-H., Kim H.-T., Kang B.-Y., Lee H.-W. (2024). Deep reinforcement learning for PID parameter tuning in greenhouse HVAC system energy Optimization: A TRNSYS-Python cosimulation approach. Expert Syst. Appl..

[53] Cheng Y. (2020). Research on intelligent control of an agricultural greenhouse based on fuzzy PID control. J. Environ. Eng. Sci..

[54] Riahi J., Vergura S., Mezghani D., Mami A. (2020). Intelligent Control of the Microclimate of an Agricultural Greenhouse Powered by a Supporting PV System. Appl. Sci..

[55] Mac T.T., Nguyen T.-D., Dang H.-K., Nguyen D.-T., Nguyen X.-T. (2024). Intelligent agricultural robotic detection system for greenhouse tomato leaf diseases using soft computing techniques and deep learning. Sci. Rep..

[56] Sun C., Qiu W. (2022). Feedback regulation system for spraying parameters based on online droplet mass deposit measurements. Int. J. Agric. Biol. Eng..

[57] Chakravarty S.P., Roy P. (2023). Quantitative feedback theory-based multi-variable robust control for soil quality improvement in a drip irrigated field. J. Process Control.

[58] Fu J., Chen C., Zhao R., Ren L. (2020). Accurate Variable Control System for Boom Sprayer Based on Auxiliary Antidrift System. J. Sens..

[59] Tao G. (2014). Multivariable adaptive control: A survey. Automatica.

[60] Wang L., Wang B. (2020). Construction of greenhouse environment temperature adaptive model based on parameter identification. Comput. Electron. Agric..

[61] Wang L., Zhang H. (2018). An adaptive fuzzy hierarchical control for maintaining solar greenhouse temperature. Comput. Electron. Agric..

[62] Boughamsa M., Ramdani M. (2018). Adaptive fuzzy control strategy for greenhouse micro-climate. Int. J. Autom. Control.

[63] Afram A., Janabi-Sharifi F. (2014). Theory and applications of HVAC control systems—A review of model predictive control (MPC). Build. Environ..

[64] Bersani C., Ouammi A., Sacile R., Zero E. (2020). Model Predictive Control of Smart Greenhouses as the Path towards Near Zero Energy Consumption. Energies.

[65] Achour Y., Ouammi A., Zejli D., Sayadi S. (2020). Supervisory Model Predictive Control for Optimal Operation of a Greenhouse Indoor Environment Coping With Food-Energy-Water Nexus. IEEE Access.

[66] Bersani C., Fossa M., Priarone A., Sacile R., Zero E. (2021). Model Predictive Control versus Traditional Relay Control in a High Energy Efficiency Greenhouse. Energies.

[67] Lin D., Zhang L., Xia X. (2020). Hierarchical model predictive control of Venlo-type greenhouse climate for improving energy efficiency and reducing operating cost. J. Clean. Prod..

[68] Mahmood F., Govindan R., Bermak A., Yang D., Al-Ansari T. (2023). Data-driven robust model predictive control for greenhouse temperature control and energy utilisation assessment. Appl. Energy.

[69] Caceres G.B., Ferramosca A., Gata P.M., Martin M.P. (2023). Model Predictive Control Structures for Periodic ON-OFF Irrigation. IEEE Access.

[70] Yu Y., Si X., Hu C., Zhang J. (2019). A Review of Recurrent Neural Networks: LSTM Cells and Network Architectures. Neural Comput..

[71] Kamilaris A., Prenafeta-Boldu F.X. (2018). A review of the use of convolutional neural networks in agriculture. J. Agric. Sci..

[72] Ojo M.O., Zahid A. (2022). Deep Learning in Controlled Environment Agriculture: A Review of Recent Advancements, Challenges and Prospects. Sensors.

[73] Castaneda-Miranda A., Castano V.M. (2017). Smart frost control in greenhouses by neural networks models. Comput. Electron. Agric..

[74] Fourati F. (2014). Multiple neural control of a greenhouse. Neurocomputing.

[75] Jung D.-H., Lee T.S., Kim K., Park S.H. (2022). A Deep Learning Model to Predict Evapotranspiration and Relative Humidity for Moisture Control in Tomato Greenhouses. Agronomy.

[76] Liu Y., Li D., Wan S., Wang F., Dou W., Xu X., Li S., Ma R., Qi L. (2022). A long short-term memory-based model for greenhouse climate prediction. Int. J. Intell. Syst..

[77] Guesbaya M., Garcia-Manas F., Rodriguez F., Megherbi H. (2023). A Soft Sensor to Estimate the Opening of Greenhouse Vents Based on an LSTM-RNN Neural Network. Sensors.

[78] Wu W., Yao B., Huang J., Sun S., Zhang F., He Z., Tang T., Gao R. (2023). Optimal temperature and humidity control for autonomous control system based on PSO-BP neural networks. IET Control. Theory Appl..

[79] Belhaj Salah L., Fourati F. (2021). A greenhouse modeling and control using deep neural networks. Appl. Artif. Intell..

[80] Cevallos G., Herrera M., Jaimez R., Aboukheir H., Camacho O. (2022). A Practical Hybrid Control Approach for a Greenhouse Microclimate: A Hardware-in-the-Loop Implementation. Agriculture.

[81] Zhang L., Shen Y. (2019). Intelligent measurement and control system of greenhouse environment based on the of hybrid incremental PID control. Basic Clin. Pharmacol. Toxicol..

[82] Castaneda-Miranda A., Castano-Meneses V.M. (2020). Smart frost measurement for anti-disaster intelligent control in greenhouses via embedding IoT and hybrid AI methods. Measurement.

[83] Ding J.-T., Tu H.-Y., Zang Z.-L., Huang M., Zhou S.-J. (2018). Precise control and prediction of the greenhouse growth environment of Dendrobium candidum. Comput. Electron. Agric..

[84] Mohamed S., Hameed I.A. (2018). A GA-Based Adaptive Neuro-Fuzzy Controller for Greenhouse Climate Control System. Alex. Eng. J..

[85] Montoya A.P., Guzman J.L., Rodriguez F., Sanchez-Molina J.A. (2016). A hybrid-controlled approach for maintaining nocturnal greenhouse temperature: Simulation study. Comput. Electron. Agric..

[86] Yan H., Acquah S.J., Zhang J., Wang G., Zhang C., Darko R.O. (2021). Overview of modelling techniques for greenhouse microclimate environment and evapotranspiration. Int. J. Agric. Biol. Eng..

[87] Xu D., Ren L., Zhang X. (2023). Predicting Multidimensional Environmental Factor Trends in Greenhouse Microclimates Using a Hybrid Ensemble Approach. J. Sens..

[88] Bouhoun Ali H., Bournet P.-E., Cannavo P., Chantoiseau E. (2018). Development of a CFD crop submodel for simulating microclimate and transpiration of ornamental plants grown in a greenhouse under water restriction. Comput. Electron. Agric..

[89] Sciuto G.L., Cammarata G., Capizzi G., Coco S. Numerical simulation of a typical bioclimate greenhouse in winter on cloudy days. Proceedings of the 2018 International Symposium on Power Electronics, Electrical Drives, Automation and Motion, SPEEDAM.

[90] Wang H., Li L., Wu Y., Meng F., Wang H., Sigrimis N.A. Recurrent Neural Network Model for Prediction of Microclimate in Solar Greenhouse. Proceedings of the 6th International-Federation-of-Automatic-Control (IFAC) Conference on Bio-Robotics (BIOROBOTICS).

[91] Villagran E.A., Baeza Romero E.J., Bojaca C.R. (2019). Transient CFD analysis of the natural ventilation of three types of greenhouses used for agricultural production in a tropical mountain climate. Biosyst. Eng..

[92] Pakari A., Ghani S. (2019). Airflow assessment in a naturally ventilated greenhouse equipped with wind towers: Numerical simulation and wind tunnel experiments. Energy Build..

[93] Ma D., Carpenter N., Maki H., Rehman T.U., Tuinstra M.R., Jin J. (2019). Greenhouse environment modeling and simulation for microclimate control. Comput. Electron. Agric..

[94] Saberian A., Sajadiye S.M. (2019). The effect of dynamic solar heat load on the greenhouse microclimate using CFD simulation. Renew. Energy.

[95] Yau J., Ji J., Wang H., Eniola O., Ibitoye F.P. (2020). Modeling of the Internal Temperature for an Energy Saving Chinese Solar Greenhouse. Eng. Technol. Appl. Sci. Res..

[96] Ben Ali R., Bouadila S., Mami A. (2020). Experimental validation of the dynamic thermal behavior of two types of agricultural greenhouses in the Mediterranean context. Renew. Energy.

[97] Jung D.-H., Kim H.S., Jhin C., Kim H.-J., Park S.H. (2020). Time-serial analysis of deep neural network models for prediction of climatic conditions inside a greenhouse. Comput. Electron. Agric..

[98] Ernesto Aguilar-Rodriguez C., Flores-Velazquez J., Ojeda-Bustamante W., Rojano F., Iniguez-Covarrubias M. (2020). Valuation of the Energy Performance of a Greenhouse with an Electric Heater Using Numerical Simulations. Processes.

[99] Gharghory S.M. (2020). Deep Network based on Long Short-Term Memory for Time Series Prediction of Microclimate Data inside the Greenhouse. Int. J. Comput. Intell. Appl..

[100] Chalill S.M., Chowdhury S., Karthikeyan R. (2021). Prediction of Key Crop Growth Parameters in a Commercial Greenhouse Using CFD Simulation and Experimental Verification in a Pilot Study. Agriculture.

[101] Liu R., Yuan S., Han L. (2022). Evaluation and Analysis on the Temperature Prediction Model for Bailing Mushroom in Jizhou, Tianjin. Agriculture.

[102] Zhao X., Han Y., Lewlomphaisarl U., Wang H., Hua J., Wang X., Kang M. (2022). Parallel Control of Greenhouse Climate With a Transferable Prediction Model. IEEE J. Radio Freq. Identif..

[103] Yang Y., Gao P., Sun Z., Wang H., Lu M., Liu Y., Hu J. (2023). Multistep ahead prediction of temperature and humidity in solar greenhouse based on FAM-LSTM model. Comput. Electron. Agric..

[104] Habashi W.G. (2023). A Path to Enabling a Wider Use of Controlled-Accuracy 3D CFD in Industry and Academia. Int. J. Unconv. Comput..

[105] Yang Y., Gao P., Sun Z., Wang H., Lu M., Liu Y., Hu J. (2019). Optimizing the 3D Distributed Climate inside Greenhouses Using Multi-Objective Optimization Algorithms and Computer Fluid Dynamics. Energies.

[106] Katzin D., van Mourik S., Kempkes F., van Henten E.J. (2020). GreenLight—An open source model for greenhouses with supplemental lighting: Evaluation of heat requirements under LED and HPS lamps. Biosyst. Eng..

[107] Huang J., Sedano F., Huang Y., Ma H., Li X., Liang S., Tian L., Zhang X., Fan J., Wu W. (2016). Assimilating a synthetic Kalman filter leaf area index series into the WOFOST model to improve regional winter wheat yield estimation. Agric. For. Meteorol..

[108] Ma H., Malone R.W., Jiang T., Yao N., Chen S., Song L., Feng H., Yu Q., He J. (2020). Estimating crop genetic parameters for DSSAT with modified PEST software. Eur. J. Agron..

[109] Boote K.J., Seepaul R., Mulvaney M.J., Hagan A.K., Bashyal M., George S., Small I., Wright D.L. (2021). Adapting the CROPGRO model to simulate growth and production of *Brassica carinata*, a bio-fuel crop. Glob. Chang. Biol. Bioenergy.

[110] Wu A., Song Y., van Oosterom E.J., Hammer G.L. (2016). Connecting Biochemical Photosynthesis Models with Crop Models to Support Crop Improvement. Front. Plant Sci..

[111] Graefe J., Yu W., Koerner O. (2022). A Photosynthetic Light Acclimation Model Accounting for the Effects of Leaf Age, Chlorophyll Content, and Intra-Leaf Radiation Transfer. Front. Plant Sci..

[112] Nomura K., Yasutake D., Kaneko T., Iwao T., Okayasu T., Ozaki Y., Mori M., Kitano M. (2021). Long-term estimation of the canopy photosynthesis of a leafy vegetable based on greenhouse climate conditions and nadir photographs. Sci. Hortic..

[113] Chen N., Li X., Shi H., Hu Q., Zhang Y., Sun Y., Song F. (2021). Simulation of maize crop growth using an improved crop model considering the disintegrated area of biodegradable film. Field Crops Res..

[114] Shirley R., Pope E., Bartlett M., Oliver S., Quadrianto N., Hurley P., Duivenvoorden S., Rooney P., Barrett A.B., Kent C. (2020). An empirical, Bayesian approach to modelling crop yield: Maize in USA. Environ. Res. Commun..

[115] Ntakos G., Prikaziuk E., ten Den T., Reidsma P., Vilfan N., van der Wal T., van der Tol C. (2024). Coupled WOFOST and SCOPE model for remote sensing-based crop growth simulations. Comput. Electron. Agric..

